# Systematic Review of Genetic Modifiers Associated with the Development and/or Progression of Nephropathy in Patients with Sickle Cell Disease

**DOI:** 10.3390/ijms25105427

**Published:** 2024-05-16

**Authors:** Veerle Labarque, Emmanuel Chide Okocha

**Affiliations:** 1 Department of Pediatric Hemato-Oncology, University Hospitals Leuven, 3000 Leuven, Belgium; 2 Center for Molecular and Vascular Biology, KU Leuven, 3000 Leuven, Belgium; 3 Haematology Department, Faculty of Basic Clinical Sciences, College of Health Sciences, Nnamdi Azikiwe University, Nnewi PMB 5025, Anambra State, Nigeria

**Keywords:** sickle cell disease, nephropathy, albuminuria, kidney failure, decreased glomerular filtration, hyposthenuria, acidification deficit

## Abstract

Sickle cell nephropathy (SCN) is a common complication of sickle cell disease (SCD) that significantly contributes to morbidity and mortality. In addition to clinical and life-style factors, genetic variants influence this risk. We performed a systematic review, searching five databases. Studies evaluating the effect of genetic modifiers on SCN were eligible. Twenty-eight studies (fair-to-good quality) were included: one genome-wide association study, twenty-six case-control studies, and one article combining both approaches. *APOL1* was significantly associated with albuminuria and hyperfiltration in children and with worse glomerular filtration in adults. On the other hand, alpha-thalassemia protected patients against albuminuria and hyperfiltration, while *BCL11A* variants were protective against albuminuria alone. The *HMOX1* long GT-tandem repeat polymorphism led to a lower glomerular filtration rate. No modifiers for the risk of hyposthenuria were identified. A genome-wide association approach identified three new loci for proteinuria (*CRYL1*, *VWF*, and *ADAMTS7*) and nine loci were linked with eGFR (*PKD1L2*, *TOR2A*, *CUBN*, *AGGF1*, *CYP4B1*, *CD163*, *LRP1B*, *linc02288*, and *FPGT-TNNI3K/TNNI3K*). In conclusion, this systematic review supports the role of genetic modifiers in influencing the risk and progression of SCN. Incorporating and expanding this knowledge is crucial to improving the management and clinical outcomes of patients at risk.

## 1. Introduction

Sickle cell disease (SCD) is a paradigmatic example of the intricate interplay between genetic predisposition and life-style, culminating in a spectrum of clinical manifestations, among which nephropathy holds significant prominence. Advances in diagnosis, comprehensive care, and therapeutic strategies have greatly improved the lifespan of individuals with SCD. Yet, chronic complications contribute significantly to morbidity and mortality, and due to limited improvement in management and treatment, sickle cell nephropathy (SCN) is a major contributor compared to other SCD-associated complications [1].

Long-term disruption of blood flow through the renal medulla, caused by sickle-shaped red blood cells in this hypoxic, hypertonic and acidotic environment, contributes to kidney damage in SCD [2]. However, evolving evidence suggests that the exposure of renal cells (both renal tubular cells and podocytes) to cell-free heme and hemoglobin (Hb) due to intra-vascular hemolysis also plays a role in the development of SCN [3,4]. 

Nephropathy in SCD encompasses a diverse array of renal complications, and patients of all ages may be affected by signs or symptoms of SCN. The first manifestation of kidney disease in SCD is impaired concentrating ability (i.e., hyposthenuria), which already appears as early as infancy. As demonstrated in the Pediatric Hydroxyurea Phase III Clinical Trial (BABY HUG), hyperfiltration also starts at a very young age [5] and continues well into young adulthood in almost all patients. In addition, 4.5–27% of children with SCD develop albuminuria [6,7], and if left untreated, the patient will progress to a decreased glomerular filtration rate (GFR), ultimately culminating in kidney failure in a subset of patients at a median age of 23 years [7,8,9]. Four to twelve percent of adults with SCD progress to kidney failure [1,8] but decreased GFR and kidney failure may already occur during childhood/adolescence [10]. Other manifestations include hemoglobinuria [11] and hematuria [12,13]. 

While the hallmark of SCD lies in the presence of the hemoglobin S (HbS) variant, it is increasingly recognized that the genetic landscape extends beyond the *hemoglobin subunit beta* (*HBB*) gene, with various genetic modifiers exerting considerable influence on the clinical phenotype of affected individuals. These genetic modifiers encompass a myriad of genetic variants, including single nucleotide polymorphisms (SNPs), structural variants, and alterations in gene expression profiles.

Considering the growing recognition of genetic modifiers as key determinants of phenotypical severity in SCD, this systematic review aims to provide a comprehensive overview of the genomic landscape associated with SCN. By synthesizing existing evidence from genetic studies, functional genomics analyses, and clinical investigations, we seek to elucidate the role of genetic modifiers in the pathogenesis of SCN. The discussion explores the potential implications of genetic variants and molecular mechanisms identified in the review for understanding disease pathogenesis, risk stratification, prognostication, clinical management, and future research directions on therapeutic targets.

## 2. Materials and Methods

### 2.1. Search Strategy

This review is designed following the PRISMA 2020 guidelines (Supplementary Table S2) [14]. We developed a comprehensive literature search strategy in the following bibliographic databases: MEDLINE/PubMed, Elsevier databases (EMBASE (https://www.embase.com (accessed on 4 July 2023), Scopus), Web of Science (WoS; core collection), and CENTRAL (Cochrane). A combination of MeSH or Emtree terms as well as free-text terms within the concepts ‘sickle cell disease’, ‘genetic polymorphism’, and ‘kidney disease’ was used to identify all citations (primary literature and reviews) on genetic modifiers of the development or progression of SCN. The reference lists of relevant articles and reviews were hand-searched for additional citations. Curated collections of published genome-wide association study (GWAS) studies were accessed through GWAS Central. The full search strategy can be found in Supplementary Table S3. The protocol for this systematic review was registered on PROSPERO and can be accessed at https://www.crd.york.ac.uk/prospero/display_record.php?ID=CRD42023410466.

### 2.2. Study Selection

Any original genetic research study using human samples was eligible for inclusion in this review. We included GWAS and NGS-based studies as well as replication studies (incl. cohort and case-level studies) for validation. Reviews were only considered for their reference list(s). Editorials and letter-format papers were considered for the possibility that they reported small-scale studies. Articles in a language other than English were excluded.

All citations identified from the rigorous literature search were uploaded on the Rayyan app [15] as a single review for screening. Duplicates from the different electronic databases were excluded by Rayyan. The titles and abstracts of the remaining articles were screened by two authors independently. Decisions made for each article were recorded in Rayyan, and any disagreements were resolved by consensus. The full text of these potentially eligible articles was retrieved and further assessed by the same investigators for eligibility and inclusion in the review. Articles that passed the screening phase were imported into a single electronic library using Zotero (reference manager) software (Version 6.0.36) and carried forward for data extraction. Reference lists of included full-text articles were searched for the retrieval of any additional studies that had been missed.

### 2.3. Data Extraction

Microsoft Excel (Microsoft 365 MSO Version 2401 Build 16.0.17231.20304) was used to extract data from the included full-text articles for quality assessment, evidence synthesis, and to tabulate the results. 

Extracted information included the following: i. study details (title, authors, year of publication); ii. study population, participant demographics; iii. phenotype studied; iv. gene and genetic variant studied; v. study design; vi. outcome studied, such as effect size (beta-values, odds ratio (OR), hazard ratio (HR)), frequencies or means in different groups; vii. statistics (method, multiple correction, adjusted covariates, *p*-value). If multiple models of inheritance were investigated, data for all models were extracted.

### 2.4. Quality Assessment

Quality rating was performed using Q-Genie for genetic association studies [16] or the Newcastle Ottawa Quality Assessment Scale for non-randomized (case-control, cohort) studies. The quality of the included studies was assessed independently by two authors; any discrepancies were resolved by consensus. According to the Q-Genie rating, scores ≤35 indicate poor-quality studies, >35 and ≤45 indicate studies of moderate quality, and >45 indicate good-quality studies. Case-control and cohort studies were rated from 0 to 9. A score of 0–2 was judged as poor quality, 3–5 as fair quality, and 6–9 as good quality.

### 2.5. Ethical Considerations

This review adhered to ethical standards and guidelines for conducting systematic reviews and meta-analyses. All data were obtained from publicly available sources, and no human subjects or identifiable patient data were involved in the study. To maintain academic integrity and to respect intellectual property rights, we ensured proper citation and acknowledgment of the original sources.

## 3. Results

### 3.1. Identification and Description of Included Studies

**Figure 1:**
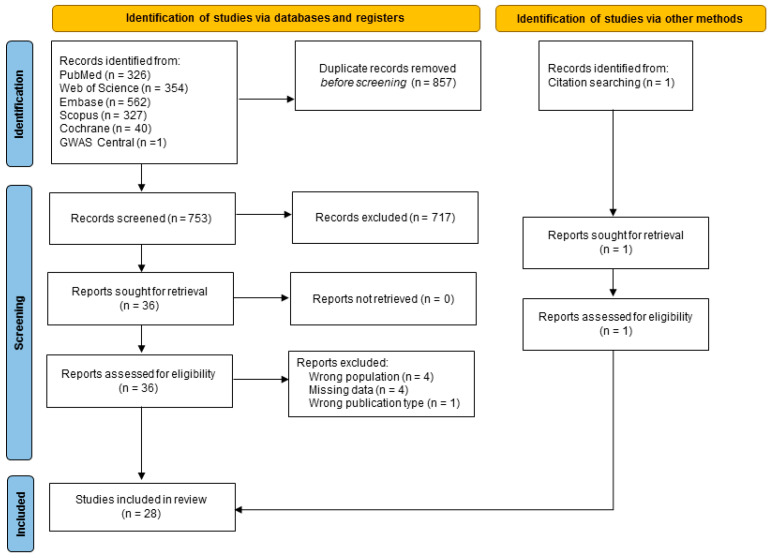
Flow diagram of the screening process. GWAS—genome-wide association study.

The systematic search yielded 1610 articles. Duplicate articles were removed, resulting in a total of 753 articles. By screening titles and abstracts, we identified 36 studies for full-text review. Nine studies were excluded because of the wrong population (n = 4), missing data to analyze the outcome (n = 4), or the wrong publication type (n = 1). Finally, one additional article was retrieved by revising the reference lists. Thus, 28 studies were included in the systematic review: 1 GWAS [17] and 26 case-control studies [9,18,19,20,21,22,23,24,25,26,27,28,29,30,31,32,33,34,35,36,37,38,39,40,41,42]. One article was set up as a GWAS to evaluate the GFR, but as a case-control study for the investigation of albuminuria [43]. An overview of the included studies and their properties is available in the Supplementary Table S1. A flow diagram displaying the screening process is also included as per guidelines set by the Preferred Reporting Items for Systematic Reviews and Meta-Analyses 2000 (PRISMA 2020) (Figure 1) [14]. These studies collectively investigated the role of genetic modifiers in the development and/or progression of nephropathy in patients with SCD.

### 3.2. Hyposthenuria and Acidification Deficit

Two case-control studies evaluated the effect of alpha-thalassemia (α^−3.7^ or α^−4.2^ allele), HbS haplotypes, or genetic variants in *BCL11A* or *NPRL3* on the occurrence of hyposthenuria [24,41]. Ndour et al. included both children and adults with homozygous sickle cell anemia (SCA) and found no association with alpha-thalassemia, Senegal haplotype, or variants in *BCL11A* (rs4671393) or *NPRL3* (rs11248850) [41], while Rocha et al. studied adult patients only with any SCD genotype and compared Bantu versus non-Bantu or Central African Republic (CAR) versus non-CAR haplotype. They could not find any association with hyposthenuria, either [24]. 

### 3.3. Proteinuria

#### 3.3.1. Results from GWAS Studies

A single GWAS evaluating the risk of proteinuria was included in this systematic review [17]. It included adults only, with any SCD genotype. Variants in the *CRYL1* (rs9315599) and *VWF* (rs2238104) gene were significantly associated with a higher risk of proteinuria (*CRYL1*: OR 3.52 (*p* = 7.26 × 10^−7^) and OR 2.91 (*p* = 0.00145) in the Outcome Modifying Genes in the SCD (OMG) cohort and Walk-Treatment of Pulmonary Hypertension and Sickle Cell Disease with Sidenafil Therapy (Walk-PHaSST) cohort, respectively; *VWF*: OR 2.08 (*p* = 0.000682) and OR 2.66 (*p* = 0.000105), respectively). A variant in *ADAMTS7* (rs3743057) was protective against the development of proteinuria (OR 0.38 (*p* = 8.64 × 10^−7^) and OR 0.62 (*p* = 0.04) in the OMG and Walk-PHaSST cohorts, respectively). 

#### 3.3.2. Results from Case-Control Studies

In addition, 21 of the 27 case-control studies evaluated the influence of various genetic variants on the risk of albuminuria and/or proteinuria: *APOL1* (n = 10), *MYH9* (n = 2), *HMOX1* (n = 9), alpha-thalassemia (n = 9), *BCL11A* (n = 3), *NPRL3* (n = 1), Duffy (n = 5), *eNOS* (n = 2), HbS haplotypes (n = 3), and others (n = 6). Studies varied in the age range of patients (children, adults, or both), SCD genotypes, outcome measures (albumin-to-creatinine ratio (ACR), quantitative albuminuria concentration, dipstick), and adjusted covariates. An overview of these studies, the significant results, and the effect size (when retrievable) are represented in Table 1. 

Ten studies investigated the role of *APOL1* in the development of albuminuria [9,23,26,29,30,33,36,37,39,43]. Of these, five studies included only children (HbSS in n = 3 [36,39,43], HbSS and HbS/beta^0^-thalassemia in n = 2 [33,37]); four other studies included only adults (all genotypes in n = 3 [23,26,29], HbSS and HbS/beta^0^-thalassemia in n = 1 [30]). A single study included homozygous SCA patients of all ages [9]. Most studies revealed the *APOL1* high-risk genotype (HRG) as a risk factor for albuminuria (ACR > 30 mg/g or >3 mg/mmol or 1+ on dipstick) [9,26,29,33,37,39,43]. Saraf et al. found a trend but no significant association in multivariate models between *APOL1* HRG and albuminuria [30]. Interestingly, Kormann et al. did not find an increased risk of albuminuria in a cross-sectional analysis of a cohort of adults with SCD and *APOL1* HRG [29]. However, they found that *APOL1* HRG was associated with worse albuminuria in an age-dependent manner. The study by Geard et al., including both children and adults, demonstrated an association with an ACR ≥3 mg/mmol but not with albuminuria >300 mg/L or crude albuminuria [9]. In addition, Schaefer et al. showed an association between ACR ≥ 30 mg/g in children with at least one *APOL1* G1 allele (rs73885319), while at least one *APOL1* G2 allele (rs71785313) was protective in their cohort [43]. Similarly, one *APOL1* G1 allele (rs73885319) was associated with proteinuria in adults [23]. A protective effect of one G2 allele against albuminuria in children was also demonstrated by Ngo-Bitoungui et al. [36].

Secondly, two variants in *HMOX1* were investigated: three studies evaluated the effect of the length of *HMOX1* GT-dinucleotide repeats [9,36,39] and six studies evaluated an SNP (rs743811) [9,26,29,34,36,37]. *HMOX1* GT-dinucleotide-long repeats were not associated with albuminuria in SCA patients. The results from studies evaluating the rs743811 variant were less conclusive. Saraf et al. found that the risk for albuminuria was increased in adults (any SCD genotype) from the University of Illinois (UIC) cohort with at least one copy of the C allele (beta 2.3, *p* = 0.014), but this could not be confirmed in the Walk-PHaSST cohort [26] or by Kormann et al. [29]. Interestingly, a study by Belisario et al. showed a lower risk of albuminuria in children with HbSS and at least one copy of the C allele (HR 0.51, *p* = 0.002) [34], but this was also not confirmed in two other studies (including SCA only or HbSS and HbS/beta^0^-thalassemia) [36,37]. Moreover, a study including patients of all ages with HbSS did not find an association between the C allele and albuminuria [9]. 

Next, the role of HbS haplotypes was investigated in three studies [18,21,41]. The Xmn1 polymorphism (*HBG2* rs7482144, i.e., Senegal haplotype) was protective against an ACR ≥ 30 mg/g in adults and children with homozygous SCA (OR 0.22, *p* = 0.035) but not against proteinuria >200 mg/g creatinine [41]. On the other hand, there was no association between the Bantou HbS haplotype and an ACR >20 mg/g [21] or the CAR haplotype and ACR ≥ 300 mg/g in adults with homozygous SCA [18].

We also included nine studies that evaluated the effect of co-inheritance of alpha-thalassemia on albuminuria. Most studies (n = 7) evaluated the most common α^−3.7^ allele [9,18,30,33,34,37,41]. However, one study additionally evaluated the α^−4.2^, α^-SEA^, α^-FIL^, α^-MED^, and α^−20.5^ deletions [40], and another one evaluated both the α^−3.7^ and α^−4.2^ alleles [21]. Studies including adults only (n = 3) concluded that alpha-thalassemia was protective against the development of albuminuria in patients with HbSS or HbS/beta^0^-thalassemia [18,21,30]. On the other hand, studies that included only children (HbSS in n = 1 [34]; HbSS or HbS/beta^0^-thalassemia in n = 2 [33,37]) found an association between alpha-thalassemia and less albuminuria only in patients with HbSS (HR 0.59, *p* = 0.047). When evaluating both children and adults together, alpha-thalassemia was protective against an ACR > 300 mg/g (*p* = 0.034 or 0.009 in a recessive or co-dominant model) but not against less-pronounced albuminuria [9,40,41]. Moreover, the protective effect could not be demonstrated in patients with compound heterozygous HbSC disease [40]. 

Furthermore, a variant in *NPRL3* (rs11248850) was protective against ACR ≥ 30 mg/g in a single study of children and adults with homozygous SCA, if in combination with an α^−3.7^ allele (OR 0.087, *p* = 0.029), but it was not protective against more pronounced proteinuria or tubular proteinuria [41].

In addition, variants in three other genes (rs6465825 in *TMEM60*, rs6795744 in *WNT7A*, and rs10951509 in *ELMO1*) were also each identified as protective against ACR ≥ 30 mg/g. *TMEM60* (beta −0.4691, *p* = 0.0234) and *WNT7A* (beta −0.5952, *p* = 0.0373) were evaluated in a pediatric cohort of homozygous SCA patients [36], while *ELMO1* (beta −0.39, *p* = 0.048) has been studied in adults with any SCD genotype [38].

Moreover, two variants in *BCL11A* (rs4671393 and rs1427407) were studied. The rs4671393 variant was significantly protective against the development of ACR ≥ 30 mg/g in patients of all ages with homozygous SCA (OR 0.27, *p* = 0.043) but not with more pronounced proteinuria or tubular proteinuria [41]. The wild-type allele (T) of the latter variant (rs1427407) was also associated with a higher odds of ACR ≥ 30 mg/g both in children and adults with HbSS or HbS/beta^0^-thalassemia (OR 2.3, *p* = 0.0077) [30,37], but significance was lost after adjusting not only for age and sex but also for diabetes, systolic blood pressure, use of hydroxyurea, treatment with an ACE inhibitor, and body mass index [30].

Two studies on *MYH9* included only adults with any SCD genotype and investigated a total of nine variants [23,26]. None were conclusively associated: five out of the nine variants were associated with an increased risk in one study but not in the other; for four of the nine variants, no association could be found in either study. 

Additionally, three variants in *eNOS* were evaluated. In contrast to the study by Tantawy et al. [27], reporting that the shorter tandem repeat polymorphism in intron 4 (eNOS4a) was significantly more frequent in children with HbSS or HbS/beta^0^-thalassemia and albuminuria (ACR ≥ 30 mg/g) (*p* = 0.006), an association could not be demonstrated by Schaefer et al. in children with homozygous SCA [43]. In addition, Schaefer et al. did not find an association between albuminuria and the rs1799983 or rs2070744 variants in *eNOS* [43]. 

We also included five studies investigating the effect of a Duffy-negative genotype on the presence of albuminuria. Schaefer et al. found that a Duffy-positive genotype had a protective effect against albuminuria in children with homozygous SCA (OR 0.29, *p* = 0.046) [43]. Similarly, Afenyi-Annan et al. reported an increased risk of proteinuria (on dipstick) in Duffy-negative adults with homozygous SCA (OR 3.1, *p* = 0.013) [20], which, however, could not be confirmed in two other studies evaluating the risk of an ACR > 200 mg/g in adults with homozygous SCA [22] or the risk of an ACR > 30 mg/g in adults with any SCD genotype [25]. Furthermore, no effect could be demonstrated in a study that included children with any SCD genotype [28]. 

Finally, five studies evaluated genetic modifiers of time to development of albuminuria. The *APOL1* HRG was associated with an earlier onset of albuminuria in children with SCD (HR 4.7, *p* = 0.0003; beta 1.25, *p* = 0.001) [33,37], whereas an *SNX* (beta 0.67, *p* = 0.048) [37], a *PARTICL* (beta 0.56, *p* = 0.024) [37], or a *GATM* variant (beta 0.61, *p* = 0.028) [37] was associated with a later onset of albuminuria in children with HbSS or HbS/beta^0^-thalassemia. Co-inheritance of alpha-thalassemia also led to a delayed onset of albuminuria in patients (all ages) with HbSS [21] and the presence of at least one C allele of the *HMOX1* variant was associated with a decreased cumulative risk of albuminuria in children with HbSS (*p* = 0.028) [34]. Studying children with either HbSS or HbS/beta^0^-thalassemia, Rashkin et al. could not confirm the protective effect of a C allele of the *HMOX1* variant or an α^−3.7^ deletion [37]. In addition, the rs1427407 variant in *BCL11A* had no influence on the age at onset of albuminuria [37]. 

### 3.4. Changes in Glomerular Filtration Rate

#### 3.4.1. Results from GWAS Studies

Two GWAS studies investigated genetic modifiers of the GFR in patients with SCD. Schaefer et al. included only children with homozygous SCA and identified variants in six genes as related to estimated GFR (eGFR) [43]. Variants in *PKD1L2* (rs76056952), *TOR2A* (rs114990094) and *CUBN* (rs111265129) were protective against elevated eGFR (*PKD1L2*: beta −0.28, *p* = 0.00011; *TOR2A*: beta −0.26, *p* = 0.00037 and *CUBN*: beta −0.23, *p* = 0.00186), while variants in *AGGF1* (rs72765108), *CYP4B1* (rs12094024) and *CD163* (rs61729510) were associated with a higher risk of hyperfiltration (*AGGF1*: beta 0.28, *p* = 0.00011; *CYP4B1*: beta 0.22, *p* = 0.00256 and *CD163*: beta 0.23, *p* = 0.00237) (Table 2).

Another GWAS, in adults with SCD, identified variants in three additional genes as associated with eGFR (Table 2) [17]: rs1968911 in *LRP1B*, rs4903539 in *linc02288*, and rs7526762 in *FPGT-TNNI3K/TNNI3K*. These associations were independent of the *APOL1* genotype. Moreover, the rs9315599 variant in *CRYL1* was found to be linked with a rapid decline in eGFR (OR 3.76, *p* = 0.004), whereas patients with the variant in *LRP1B* were less likely to suffer a rapid renal decline (OR 0.41, *p* = 0.01).

#### 3.4.2. Results from Case-Control Studies

Nineteen case-control studies investigated the effect of genetic variants on the risk of hyperfiltration, decreased eGFR (defined as GFR <60 mL/min/1.73m^2^), chronic kidney disease (CKD) stage, kidney failure (defined as GFR <15 mL/min/1.73m^2^ or need for replacement therapy), or end-stage renal disease (ESRD) (if cut-off for GFR was not defined in the study): *APOL1* (n = 6), *MYH9* (n = 1), *HMOX1* (n = 6), alpha-thalassemia (n = 6), *BCL11A* (n = 2), Duffy genotype (n = 3), HbS haplotypes (n = 2), and others (n = 6). An overview of these studies, the significant results, and the effect size (when retrievable) are represented in Table 2.

First, six studies reported the effect of *APOL1* variants. In children with homozygous SCA, *APOL1* HRG was significantly associated with eGFR and increased the risk of hyperfiltration (beta 2, *p* = 0.001) [39]. However, the HRG was not associated with decreased eGFR in children [39]. In contrast, *APOL1* HRG was significantly associated with eGFR, the CKD stage, and kidney failure in adults with any SCD genotype. In adults with HbSS or HbS/beta^0^-thalassemia, *APOL1* HRG also increased the risk of CKD, defined as a 50% reduction in eGFR or need for replacement therapy [26,29,30] but not the likelihood of a decreased eGFR (<60 mL/min/1.73 m^2^) [30]. In addition, *APOL1* HRG was associated with a more rapid progression of CKD [30]. Yet, Geard et al. could not confirm the association with eGFR when studying patients with HbSS of all ages together [9]. Evaluating the effect of the *APOL1* G1 variant, Ngo-Bitoungui et al. found a significant association between the rs71785313 variant and eGFR in children with SCA (beta 0.890305, *p* = 0.0461) [36], but this was not confirmed in adults with SCD [23]. No association could be found between the rs60910145 *APOL1* G1 variant and eGFR [36]. On the other hand, *APOL1* G2 was associated with a significantly worse eGFR than *APOL1* G1 in adults with SCD (*p* = 0.045) [29]. This finding was not confirmed in children with SCA, and there was no difference between *APOL1* G2 and G0 [36].

Secondly, a single study in adults (any SCD genotype) evaluated eight different variants in *MYH9* and demonstrated an association between the rs11912763 or rs933224 variant and GFR after adjusting for age (*p* = 0.01 and *p* = 0.004, respectively) [23]. 

Six studies evaluated the effect of the co-inheritance of alpha-thalassemia on glomerular filtration. This was protective against hyperfiltration (HR 0.45, *p* = 0.006; OR 0.3632, *p* = 0.01007) as well as against reduction in eGFR (beta −11.813; *p* = 0.006) in children with HbSS [34,36]. However, no effect on the eGFR, risk of decreased GFR, or kidney failure could be seen in studies evaluating patients of all ages or adults only with HbSS, HbS/beta^0^-thalassemia, or HbSC disease [9,30,40,41]. 

Next, six studies reported on three variants in *HMOX1*. In children with SCA [39] and in adults with any SCD genotype [26,35], *HMOX1* GT-dinucleotide long repeats were associated with a lower eGFR. In children, this resulted in a lower prevalence of hyperfiltration (beta −1.7; *p* = 0.02), but not in a higher risk of decreased eGFR [39]. In contrast, Geard et al. did not find an association in patients of all ages with homozygous SCA [9]. The effect of the C allele of the rs743811 variant was less consistent, especially in adults. Saraf et al. reported that this risk allele was associated with a lower eGFR (beta −11.7, *p* = 0.002), worse CKD stage (OR 3, *p* = 0.00013), and higher risk of kidney failure (OR 10, *p* = 0.00032) in adult patients from the UIC cohort after adjusting for *APOL1* variants, but not in the Walk-PHaSST cohort [26]. Kormann et al. could not confirm the association with lower eGFR [29]. This was also not associated with a lower eGFR in children with HbSS [34]. Only one study evaluated the effect of the rs2071746 variant in adults with SCA and found a significant association with lower eGFR (*p* = 0.009) [35].

Additionally, the effect of a Duffy-negative genotype on glomerular filtration was evaluated in adult patients with HbSS [20,22], HbS/beta^0^-thalassemia [25], or with any SCD genotype [25] using different methods. Evaluation of the glomerular filtration by measuring cystatin C levels or using the Hoek formula demonstrated higher cystatin C levels and lower eGFR in Duffy-positive patients, including all SCD genotypes [25]. No association could be found when evaluating only HbSS patients [20,22] or evaluating the presence of ESRD [25].

Furthermore, three studies evaluating the effect of six different variants in *BMPR1B* on eGFR were included in this review. Two of these studies included only adults, either with HbSS [35] or with any SCD genotype [26]. None of these studies could find an association between a polymorphism and eGFR. However, Nolan et al. evaluated patients of all ages with HbSS and found that some variants (rs17022863, rs2240036, rs4145993, rs1434549) were significantly associated with a higher eGFR (*p* = 0.011; *p* = 0.0434; *p* = 0.0352; *p* = 0.0109, respectively) [19]. 

Finally, a positive association with hyperfiltration was also found in children with HbSS for a variant in *ACE* (rs4646994) (HR 1.28, *p* = 0.027) [34] and in patients of all ages with HbSS for a variant in *NPRL3* (rs11248850), if in combination with a −3.7 kb *HBA1/HBA2* deletion (OR 17.69, *p* = 0.013) [41]. A variant in *A1CF* (rs10994860) protected against hyperfiltration and renal dysfunction (beta −0.69852, *p* = 0.020), whereas a variant in *SYPL2* (rs12136063) was associated with an increased risk (beta 0.57907, *p* = 0.04208) [36]. On the contrary, the HbS haplotype (Xmn1) polymorphism (i.e., Senegal haplotype) [41], Bantu versus non-Bantu, or CAR versus non-CAR haplotype were not linked with hyperfiltration or decreased GFR in adults [24]. Similarly, Ndour et al. reported no association between the rs4671393 variant in *BCL11A* and the risk of hyperfiltration in patients with SCA [41], while Saraf et al. found no association between the rs142740 variant in *BCL11A* and the risk of decreased GFR or CKD (defined as a reduction of 50% of the eGFR or need for replacement therapy) in adults with HbSS or HbS/beta^0^-thalassemia [30].

### 3.5. Hemoglobinuria

Two case-control studies evaluated genetic modifiers of the risk for hemoglobinuria. Saraf et al. studied the association of variants in *APOA1* (rs11216132), *APOL1* (HRG) or *HMOX1* (rs743811) in adult SCD patients [26,38]. This was defined as fewer than two red blood cells per high-power field on two consecutive urinalyses (UIC cohort) or fewer than five red blood cells per high-power field on one urine sample (Walk-PHaSST cohort). They found that *APOL1* HRG was associated with a significantly increased risk of hemoglobinuria (OR 2.6, *p* = 0.003 in a recessive model and *p* = 0.01 in an additive model) [26]. No increased risk was found for patients with *APOA1* [38] or *HMOX1* variants [26]. 

### 3.6. Quality Assessment

Twenty-one of the case-control studies were of good quality [9,18,19,20,21,23,24,25,26,29,30,31,33,34,36,37,38,39,40,41,43] and six studies were rated as of fair quality [22,27,28,32,35,42]. No studies of poor quality were included in this review. The quality of one GWAS was assessed as good [17], and the other as moderate [43]. Of note, the study by Schaefer et al. was rated in two different ways, according to the double set-up of this study [43]. 

## 4. Discussion

This systematic review elucidates the significant influence of genetic modifiers on the development and progression of nephropathy in SCD. Genetic variants, including apolipoprotein L1 (*APOL1*), myosin-9 (*MYH9*), alpha-thalassemia (*HBA1/HBA2* deletions), those affecting hemoglobin regulation (e.g., *BCL11A*), heme degradation (*HMOX1*) and the renin–angiotensin system genes (e.g., *ACE*), were identified as key risk factors of albuminuria and/or glomerular filtration in SCD populations. These modifiers exert their effects through diverse molecular pathways, including endothelial dysfunction, inflammation, oxidative stress, rheological features, and podocyte injury, contributing to the pathogenesis of renal complications in SCD. However, the exploration of genetic modifiers associated with nephropathy in SCD extends beyond well-established variants, encompassing additional factors such as hyposthenuria and acidification deficit. Researchers, indeed, also tested genetic variants that are already associated with kidney problems in non-SCD populations [38,44,45,46,47,48,49].

### 4.1. Proteinuria, Decreased Glomerular Filtration Rate and Kidney Failure

Albuminuria emerges as a crucial clinical marker, as delineated by various studies [6,18,50]. Moreover, the presence of albuminuria in pediatric SCA patients, as demonstrated in several papers [33,37,39], suggests an early onset of renal complications and underscores the importance of close monitoring and early intervention to mitigate disease progression. Incorporating insights from the manuscripts by Kormann et al., Zahr et al., Rashkin et al., Adebayo et al., and Schaefer et al. further expands our understanding of the complex interplay between genetic factors, clinical parameters, and renal outcomes [29,33,37,39,43]. Adebayo et al. demonstrate the association between genetic factors (*APOL1* HRG, *HMOX1* long GT repeats), clinical parameters (hemoglobin levels and age), and kidney complications in African children with SCA [39]. Additionally, genetic modifiers identified by Schaefer et al. influence white blood cell count, albuminuria, and GFR [43]. The molecular mechanisms underlying the pathogenesis of albuminuria and its progression to kidney failure in SCD are complex and multifaceted, involving dysregulation of glomerular filtration, podocyte function, and tubular reabsorption pathways. The identification of genetic modifiers influencing these pathways has significant clinical implications for risk stratification, prognostication, and therapeutic targeting. 

Genetic variants in *APOL1* have been identified as significant determinants of renal complications in individuals of African descent, such as focal segmental glomerulosclerosis, human immunodeficiency virus-associated nephropathy, and lupus nephritis [51,52,53], and have also been extensively studied in SCD. Few studies evaluated the effect of one risk allele, but they found that the presence of one *APOL1* G1 allele was associated with albuminuria in children [43] and with proteinuria in adults [23], while a single G2 allele was protective against albuminuria in children [36,43]. On the other hand, the *APOL1* HRG is clearly shown to be an important risk factor for albuminuria [9,26,29,33,37,39,43] both in children and adults with SCD, for hyperfiltration in children [39], and for worse glomerular filtration in adults [26,29,30]. Indeed, this genotype increases the risk of moderately increased albuminuria (ACR > 30 mg/g) already during childhood. Moreover, the presence of *APOL1* HRG leads to an earlier onset of albuminuria [33,37]. These findings are consistent with the higher risk of hyperfiltration in children, but also with the observation of worse glomerular filtration in adults. APOL1 is expressed in several kidney cell types, but the mechanisms by which the high-risk variants cause kidney disease are not yet well understood. However, prolonged glomerular hyperfiltration likely contributes in part to the development of albuminuria [54], which ultimately leads to kidney failure [6]. 

The most important genetic factor protecting against albuminuria and hyperfiltration is the co-inheritance of alpha-thalassemia. Children with HbSS, as well as adults with HbSS or HbS/beta^0^-thalassemia, were less likely to have albuminuria if they carried at least one alpha-globin deletion [18,21,30]. Co-inheritance of alpha-thalassemia also protected against hyperfiltration in children [34,36]. Accordingly, no significant effect was observed for the risk of decreased GFR or chronic kidney failure at any age [9,30,40,41]. *HBA1/HBA2* microdeletions, indeed, improve the rheological features of red blood cells and reduce hemolysis, due to the lower erythrocyte volume, reduced intra-erythrocyte concentration of HbS, and reduced red blood cell dehydration [40]. This may lead to either decreased vaso-occlusion or hemolysis-related glomerular damage. Of note, the hematological effects of alpha-thalassemia emerge when HbF levels have fallen below 15%. This may influence the significance of associations, depending on the age of patients included in the studies. Nevertheless, results show a reno-protective effect of *HBA1/HBA2* microdeletions both in children and adults. 

In addition, two variants in *HMOX1* were studied in more than one study. In response to oxidative stress, Nrf2 (nuclear factor erythroid-2-related factor 2) regulates the transcription of *HMOX1*, which encodes for the hemoxygenase-1 protein (HO-1). Intravascular hemolysis leading to the release of free heme is a major source of oxidative stress and induces HO-1 expression in patients with SCD [55]. However, the level of transcription of *HMOX1* is modulated by a GT-tandem repeat polymorphism in its promoter: a long (GT)-repeat (>25) is associated with reduced induction [56,57] and increased toxicity by reactive oxygen species [58]. In our review, the long (GT)-repeat (rs3074372) was not found to be associated with albuminuria at any age [9,36,39]. However, this variant was associated with less hyperfiltration in children and with a worse eGFR in adults [26,35,39]. Results from different studies evaluating the effect of the rs743811 *HMOX1* variant on albuminuria or GFR were conflicting, both in children and adults [26,29,34].

Moreover, genetic variants influencing the expression of fetal hemoglobin (HbF) have also emerged as pivotal modifiers of disease severity in SCD [59,60,61], potentially by mitigating the vaso-occlusive phenomena and oxidative stress implicated in the pathogenesis of SCD-associated nephropathy. Polymorphisms in genes such as *BCL11A* and *HBS1L*-*MYB* have been identified as key regulators of HbF production, offering potential targets for therapeutic modulation and personalized management of nephropathy in SCD patients. In this review, a protective effect against the development of albuminuria at any age (ACR ≥ 30 mg/g) was found for *BCL11A* (rs4671393). Lettre et al. have, indeed, confirmed that rs4671393 is strongly associated with HbF level variation in SCD populations [62]. It is therefore not surprising that this *BCL11A* variant would be correlated with reduced morbidity. However, no effect on glomerular filtration could be demonstrated (rs4671393 and rs1427407): the risk of hyperfiltration was unchanged in patients of all ages (rs4671393), as was the risk of worse GFR in adults (rs1427407) [30,41]. Of note, hyperfiltration is more prevalent during childhood. Including adults in a study evaluating the effect of a variant (rs467139) on hyperfiltration may thus influence the results. 

In addition to the aforementioned genetic modifiers, a genome-wide association approach identified 12 new loci that were independently associated with proteinuria (*CRYL1, VWF*) or protection against proteinuria in adults (*ADAMTS7*); with protection against elevated eGFR (*PKD1L2*, *TOR2A*, *CUBN*) or higher risk of hyperfiltration in children (*AGGF1*, *CYP4B1*, *CD163*) or with decreased eGFR in adults (*LRP1B*, *linc02288*, *FPGT-TNNI3K/TNNI3K*) [17,43]. These genes are involved in pathways important to both renal function and SCD biology, and some of them have already been linked to renal disease in non-SCD populations [63,64]. They thus provide new targets for functional follow-up or treatment.

Finally, for variants in various other genes, a limited number of studies suggested or argued against an effect on albuminuria (e.g., HbS haplotypes, *NPRL3*, *TMEM60*, *WNT7A*, *ELMO1*, *eNOS*, *PARTICL*, *SNX*, *GATM,* Duffy genotype), GFR (e.g., HbS haplotypes, *NPRL3*, *BMPR1B*, *ACE*, *A1CF*, *SYPL2*, Duffy genotype) or hemoglobinuria (e.g., *APOA1*, *APOL1*, *HMOX1*), but the evidence for these variants is currently still too limited and needs to be further investigated.

Despite advances in understanding the genetic basis of albuminuria in SCD, challenges remain in translating genetic findings into clinical practice. Further research is needed to elucidate the functional significance of genetic variants, assess their utility as prognostic markers, and explore gene-environment and gene–gene interactions influencing albuminuria progression and treatment outcomes. Future studies should also consider the broader socio-economic determinants influencing disease progression and treatment response in diverse SCD populations. 

### 4.2. Hyposthenuria and Acidification Deficit

Hyposthenuria, characterized by the inability to concentrate urine, and acidification deficit, reflecting impaired renal acidification capacity, represent distinct renal abnormalities observed in individuals with SCD. These manifestations contribute to the clinical spectrum of SCN, with implications for disease progression and management. The molecular mechanisms underlying hyposthenuria and acidification deficit in SCD are multifactorial and may involve dysregulation of renal tubular function, alterations in ion transport pathways, and impaired acid–base homeostasis. Rocha et al. and Ndour et al. have investigated the role of genetic variants within the beta S-haplotypes, Senegal haplotype (Xmn1-rs7412844), alpha-thalassemia (3.7kb or 4.2 kb *HBA1/HBA2* deletion), *NPRL3*-rs11248850, and *BCL11A*-rs4671393 in modulating hyposthenuria and acidification deficit in SCD patients, but could not reveal a role for either of these variants, in adults and in patients of all ages, respectively [24,41]. 

### 4.3. Limitations

Some limitations that may confound our conclusions are worth noting. These include the inherent heterogeneity of study designs, populations, and methodologies across included studies, which may affect the generalizability and interpretation of findings. Indeed, the most frequently studied genotypes were homozygous HbSS and HbS/beta^0^-thalassemia, known as the most severe genotypes, while patients with HbS/beta^+^-thalassemia or compound heterozygous HbSC disease were less frequently included in the studies. Also, 15 out of the 27 case-control studies did not report on the impact of gender on the effect of a genetic variant. However, the other studies adjusted for gender and thus showed that the (absence of) effect was independent of the gender. In addition, the method of measuring albuminuria varied among the studies. Most studies measured the urinary ACR quantitatively and used 30 mg/g as a cut-off, while some studies used a different cut-off value, and others assessed albuminuria or proteinuria based on a dipstick alone. However, it has already been shown that the results of a dipstick have lower sensitivity and positive predictive values, making quantitative measurements more advisable for early and reliable detection [65]. The diversity in instruments for measuring albuminuria, as well as in the definitions, may have influenced the results of the studies and thus the interpretation of the modifying effect. Also, most studies evaluated GFR using formulas based on creatinine levels. However, it is well known that other methods, e.g., cystatin C, reflect the GFR more reliably and independently of body composition [66]. Moreover, SCD patients have an increased tubular creatinine secretion, further disturbing the reliability of creatinine-based eGFR calculations [67]. As a result, the effect of variants on kidney function may have been incorrectly estimated. In addition, most studies were based on a single cross-sectional measurement, even though the Kidney Disease: Improving Global Outcomes (KDIGO) clinical practice guideline warns not to diagnose chronic kidney disease based on a single measurement [68]. Furthermore, the majority of studies evaluated variants in individual genes, while some included a genetic profile analysis. Results suggest that a genetic profile may have a stronger impact than variants in individual genes [30]. Future studies should take this into account when evaluating genetic modifiers. Additionally, the dynamic nature of genomic research may result in updates or revisions to the current understanding of genetic modifiers in SCN that are beyond the scope of this review. Finally, only two studies reported on data from a GWAS, while all other articles examined the effect of candidate genetic variants, some of which were studied in only one study. On the other hand, most of the papers included were assessed as good quality, which is a strength of this review. 

## 5. Conclusions

The identification of albuminuria as an early marker of renal involvement in SCD has significant implications for clinical management and patient care. Monitoring and early detection of albuminuria enable timely intervention and risk stratification to prevent progression to decreased glomerular filtration and kidney failure. Incorporating genetic information may further refine risk assessment and inform personalized treatment strategies for SCD patients at risk of renal complications. Moving forward, future research should focus on elucidating the mechanistic underpinnings of albuminuria in SCD, including the role of genetic modifiers and environmental factors in disease pathogenesis. Endorsed by the European Reference Network for Rare Hematological Diseases, ERN-EuroBloodNet, the International Hemoglobinopathy Research Network (INHERENT) was established with the aim of discovering novel genetic modifiers of hemoglobinopathies and validating previously reported ones through a large-scale, multi-ethnic GWAS [69]. Longitudinal studies assessing the trajectory of renal dysfunction and the efficacy of interventions are also needed to optimize clinical management and improve outcomes in SCD patients with albuminuria.

## Figures and Tables

**Table 1:** Overview of studies evaluating genetic modifiers of albuminuria.

	Gene	Rs_ID	Cytogenetic Location	EA	OA	SCD Genotype	N	Outcome	Effect	Effect Size	*p*-Value	Ref.
ADULTS	*APOL1*	rs73885319	22q12.3	G1	G0	all	521	proteinuria ^1^			0.0022	[23]
				HRG	LRG	all	152	ACR			<0.001	[29]
				HRG	LRG	all	203	ACR	beta	1.1	0.0032	[26]
				HRG	LRG	all	152	ACR > 30 mg/mmol			0.04	[29]
				HRG	LRG	all	152	PCR			0.009	[29]
	*MYH9*	rs8141189	22q12.3	A	T	all	521	proteinuria ^1^			0.001	[23]
		rs16996672		T	C	all	521	proteinuria ^1^			0.0001	[23]
		rs1557529		A	G	all	521	proteinuria ^1^			0.0019	[23]
		rs11912763		A	G	all	521	proteinuria ^1^			0.0003	[23]
	*HBA1/HBA2*	α^−3.7^	16p13.3	no D	D	SCA/Sbeta0	205	ACR ≥ 30 mg/g	OR	3	0.00058 *	[30]
		α^−3.7^		no D	D	SCA/Sbeta0	205	ACR ≥ 30 mg/g	OR	3	0.0009 **	[30]
		α^−3.7^		no D	D	SCA/Sbeta0	205	ACR ≥ 30 mg/g	OR	2.7	0.0027 ***	[30]
		α^−3.7^		D	no D	na	76	ACR ≥ 300 mg/g	frequency		0.01	[18]
		α^−3.7^ and α^−4.2^		D	no D	SCA	183	albuminuria > 20 mg/mL	frequency		0.0057	[21]
		α^−3.7^ and α^−4.2^		D	no D	SCA	182	time to albuminuria > 20 mg/mL	HR	0.56	0.021	[21]
	risk profile ^2^					SCA/Sbeta0	205	ACR ≥ 30 mg/g	OR	4.9	0.00009 *	[30]
	risk profile					SCA/Sbeta0	205	ACR ≥ 30 mg/g	OR	4.7	0.00018 **	[30]
	risk profile					SCA/Sbeta0	205	ACR ≥ 30 mg/g	OR	4.1	0.00061 ***	[30]
	*HMOX1*	rs743811	22q12.3	C	T	all	203	ACR	beta	2.3	0.014	[26]
	*BCL11A*	rs1427407	2p16.1	T	G	SCA/Sbeta0	205	ACR ≥ 30 mg/g	OR	2.3	0.0077	[30]
	*DARC*	rs2814778	1q23.2	Fy-	Fy+	SCA	249	proteinuria^1^	OR	3.1	0.013	[20]
	*ELMO1*	rs10951509	7p14.2-p14.1	A/C	G	all	299	ACR ≥ 30 mg/g	beta	−0.39	0.048	[38]
	*CRYL1*	rs9315599	13q12.11	G	C	all	516/461 ^3^	proteinuria ^1^	OR	3.52/2.91	7.26 × 10^−7^/0.00145	[17]
	*VWF*	rs2238104	12p13.31	T	G	all	524/465 ^3^	proteinuria ^1,4^	OR	2.08/2.66	0.000682/ 0.000105	[17]
	*ADAMTS7*	rs3743057	15q25.1	T	C	all	524/465 ^3^	proteinuria ^1,4^	OR	0.38/0.62	8.64 × 10^−7^/0.04	[17]
PEDIATRIC	*APOL1*	rs73885319	22q12.3	G1	G0	SCA	197	ACR ≥ 30 mg/g	OR	2.51	0.017	[43]
		rs71785313		G2	G0	SCA	413	ACR ≥ 30 mg/dl	beta	−0.12686	0.03803	[36]
		rs71785313		G2	G0	SCA	197	ACR ≥ 30 mg/g	OR	0.36	0.048	[43]
				HRG	LRG	SCA	326	ACR ≥ 30 mg/g	beta	1.2	0.04	[39]
				HRG	LRG	SCA/Sbeta0	288	ACR > 30 mg/g	frequency		0.000154	[37]
				HRG	LRG	SCA/Sbeta0	291	ACR > 30 mg/g	prevalence		0.0217	[33]
				HRG	LRG	SCA/Sbeta0	291	time to ACR > 30 mg/g	HR	4.7	0.0003	[30]
				HRG	LRG	SCA/Sbeta0	288	time to ACR > 30 mg/g	beta	1.25	0.001	[37]
	*HBA1/HBA2*	α^−3.7^	16p13.3	D	no D	SCA	555	ACR ≥ 30 mg/g	HR	0.59	0.047	[34]
		α^−3.7^		D	no D	SCA	555	cumulative risk of albuminuria			0.01	[34]
	*HMOX1*	rs743811	22q12.3	C	T	SCA	555	ACR ≥ 30 mg/g	HR	0.51	0.002	[34]
		rs743811		C	T	SCA	555	cumulative risk of albuminuria			0.013	[34]
	*BCL11A*	rs1427407	2p16.1	G	T	SCA/Sbeta0	288	ACR > 30 mg/g	frequency		0.0082	[37]
	*eNOS*		7q35–36	4a	4b	SCA/Sbeta0	51	ACR ≥ 30 mg/g	frequency		0.006	[27]
	*DARC*	rs2814778	1q23.2	Fy+	Fy-	SCA	197	ACR ≥ 30 mg/g	OR	0.29	0.046	[43]
	*TMEM60*	rs6465825	14q22.3	T	C	SCA	413	albuminuria ≥ 30 mg/dL	beta	−0.4691	0.0234	[36]
	*WNT7A*	rs6795744	3p25.1	A	G	SCA	413	albuminuria ≥ 30 mg/dL	beta	−0.5952	0.0373	[36]
	*SNX17*	rs4665972	2p23.3	T	C	SCA/Sbeta0	288	time to ACR > 30 mg/g	beta	0.67	0.048	[37]
	*PARTICL*	rs12714144	2p11.2	A	T	SCA/Sbeta0	288	time to ACR >30 mg/g	beta	0.56	0.024	[37]
	*GATM*	rs1145078	15q21.1	C	T	SCA/Sbeta0	288	time to ACR >30 mg/g	beta	0.61	0.028	[37]
ALL	*APOL1*		22q12.3	HRG	LRG	SCA	413	ACR ≥ 3 mg/mmol			0.018	[9]
	*HBA1/HBA2*	α^−3.7^	16p13.3	D	no D	SCA	413	ACR > 300 mg/g			0.034/0.009 ^§^	[9]
		α^−3.7^, α^−4.2^, α^-SEA^, α^-FIL^, α^-MED^, α^−20.5^		D	no D	SCA	358	ACR			0.00311	[40]
	*BCL11A*	rs4671393	2p16.1	G	A	SCA	162	ACR ≥ 30 mg/g	OR	0.27	0.043	[41]
	*NPRL3* ^5^	rs11248850	16p13.3	A	G	SCA	162	ACR ≥ 30 mg/g	OR	0.087	0.029	[41]
	*HBG2*	rs7482144	11p15.4	T	C	SCA	162	ACR ≥ 30 mg/g	OR	0.22	0.035	[41]

Effect and effect size were noted whenever retrievable from the original study. ACR—albumin-to-creatinine ratio, D—deletion, EA—effect allele, HR—hazard ratio, HRG—high risk genotype, L—long GT-tandem repeat, LRG—low-risk genotype, N—number of patients included in the original study, OA—other allele, OR—odds ratio, PCR—protein-to-creatinine ratio, Ref.—reference, S—short GT-tandem repeat, Sbeta0—compound heterozygous hemoglobin S/beta^0^-thalassemia, SCA—sickle cell anemia, ^1^ defined as dipstick 1+ or more, ^2^ genetic risk profile defined as SCA patients with *APOL1* HRG, without alpha-thalassemia and with wild-type *BCL11* rs1427407 allele, ^4^ proteinuria measured by dipstick (≥ 1+ or more) and quantitatively (≥30 mg/dl), ^5^ only in combination with α^−3.7^ deletion, ^3^ Outcome Modifying Genes in SCD cohort/Walk-Treatment of Pulmonary Hypertension and Sickle Cell Disease with Sidenafil Therapy cohort, ^§^ recessive/co-dominant model, * adjusted for age and sex, ** adjusted for age, sex, diabetes, systolic blood pressure, hydroxyurea therapy, angiotensin-converting-enzyme inhibitor or angiotensin receptor blocker use, body mass index, *** adjusted for age, sex, diabetes, systolic blood pressure, hydroxyurea therapy, angiotensin-converting-enzyme inhibitor or angiotensin receptor blocker use, body mass index, and urinary albumin-to-creatinine ratio.

**Table 2:** Overview of studies evaluating genetic modifiers of glomerular filtration.

	Gene	Rs_ID	Cytogenetic Location	EA	OA	Genotype	N	Outcome	Effect	Effect Size	*p*-Value	Ref.
ADULT	*APOL1*	rs73885319	22q12.3	G1	G0	all	521	GFR ^1^			0.01	[23]
		rs71785313		G2	G1	all	152	eGFR			0.045	[29]
				HRG	LRG	all	215	eGFR	beta	−11.6	0.039	[26]
				HRG	LRG	all	152	eGFR			0.008	[29]
				HRG	LRG	all	152	eGFR < 15	OR	32.3	0.003	[29]
				HRG	LRG	all	205	kidney failure ^2^	OR	6.5	0.036	[26]
				HRG	LRG	all	205	CKD stage	OR	2.6	0.022	[26]
				HRG	LRG	all	152	CKD stage			0.001	[29]
				HRG	LRG	SCA/Sbeta0	262	CKD ^3^	HR	6.3	0.00005 *	[30]
				HRG	LRG	SCA/Sbeta0	262	CKD ^3^	HR	7.8	0.00005 **	[30]
				HRG	LRG	SCA/Sbeta0	262	CKD ^3^	HR	7.4	0.0001 ***	[30]
	*MYH9*	rs11912763	22q12.3	G	A	all	521	GFR ^1^			0.01	[23]
		rs933224		G	A	all	521	GFR ^1^			0.004	[23]
	*HMOX1*	rs743811	22q12.3	C	T	all	215	eGFR	beta	−11.7	0.002	[26]
		rs743811		C	T	all	482	kidney failure ^2^	OR	10	0.00032	[26]
		rs743811		C	T	all	205	CKD stage	OR	3	0.00013	[26]
		rs3074372		LL/SL	SS	all	237	eGFR	beta	−9.2/−8.8 ^§^	0.012/0.025 ^§^	[26]
		rs3074372		LL	SL/SS	SCA	75	eGFR			0.005	[35]
		rs2071746		T	A	SCA	74	eGFR			0.009	[35]
	*DARC*	rs2814778	1q23.2	Fy−	Fy+	all	272	cystatin C			0.002	[25]
		rs2814778		Fy−	Fy+	all	272	eGFR			0.001	[25]
	risk profile ^4^					SCA/Sbeta0	205	eGFR < 60	OR	5.1	0.0083 *	[30]
						SCA/Sbeta0	205	eGFR < 60	OR	5.6	0.0091 **	[30]
						SCA/Sbeta0	205	CKD ^3^	HR	6.8	0.00004 *	[30]
						SCA/Sbeta0	205	CKD ^3^	HR	8.7	0.00005 **	[30]
						SCA/Sbeta0	205	CKD ^3^	HR	7.7	0.0003 ***	[30]
	HbS haplotype		11p15.4	CAR	non-CAR	na	57	eGFR 60–120	frequency		0.04	[24]
	*LRP1B*	rs1968911	2q22.1-q22.2	G	A	all	458/493 ^5^	eGFR	beta	−25.91/ −16.97	7.38 × 10^−5^/0.00133	[17]
	*linc02288*	rs4903539	14q24.3	G	T	all	458/493 ^5^	eGFR	beta	−20.25/ −11.76	2.62 × 10^−5^/0.00512	[17]
	*FPGT-TNNI3K/ TNNI3K*	rs7526762	1p31.1	G	A	all	453/485 ^5^	eGFR	beta	12.14/26	0.0246/ 3.23 × 10^−6^	[17]
	*CRYL1*	rs9315599	13q12.11	G	C	all	188	rapid eGFR decline ^6^	OR	3.76	0.004	[17]
	*LRP1B*	rs1968911	2q22.1-q22.2	G	A	all	189	rapid eGFR decline ^6^	OR	0.41	0.01	[17]
PEDIATRIC	*APOL1*	rs73885319	22q12.3	G1	G0	SCA	413	eGFR	beta	0.890305	0.0461	[36]
				HRG	LRG	SCA	326	log(eGFR)	beta	0.6	<0.00001	[39]
				HRG	LRG	SCA	326	hyperfiltration ^7^	beta	2	0.001	[39]
	*HBA1/HBA2*	α^−3.7^	16p13.3	D	no D	SCA	540	eGFR	beta	−11.813	0.006	[34]
		α^−3.7^		D	no D	SCA	540	eGFR ≥ 140	HR	0.45	0.006	[34]
		α^−3.7^		2 D	1/no D	SCA	413	eGFR < 90 or hyperfiltration ^8^	OR	0.3632	0.01007	[36]
	*HMOX1*	rs3074372	22q12.3	LL/SL	SS	SCA	326	log(eGFR)	beta	−0.3	0.03	[39]
		rs3074372		LL/SL	SS	SCA	326	hyperfiltration ^7^	beta	−1.7	0.02	[39]
	*A1CF*	rs10994860	10q11.23	T	C	SCA	413	eGFR	beta	−0.69852	0.0202	[36]
	*SYPL2*	rs12136063	1p13.3	A	G	SCA	413	eGFR	beta	0.57907	0.04208	[36]
	*ACE*	rs4646994	17q23.3	DI/II	DD	SCA	540	eGFR ≥ 140	HR	1.28	0.027	[34]
	*PKD1L2*	rs76056952	16q23.2	G/T	C	SCA	187	eGFR	beta	−0.28	0.00011	[43]
	*AGGF1*	rs72765108	5q13.3	A/T	G	SCA	187	eGFR	beta	0.28	0.00011	[43]
	*TOR2A*	rs114990094	9q34.11	A/C/T	G	SCA	187	eGFR	beta	−0.26	0.00037	[43]
	*CUBN*	rs111265129	10p13	C	T	SCA	187	eGFR	beta	−0.23	0.00186	[43]
	*CYP4B1*	rs12094024	1p33	C	A	SCA	187	eGFR	beta	0.22	0.00256	[43]
	*CD163*	rs61729510	12p13.31	T	C	SCA	187	eGFR	beta	0.23	0.00237	[43]
ALL	*BMPR1B*	rs17022863	4q22.3	G	A	SCA	1140	eGFR			0.011	[19]
		rs2240036		C	T	SCA	1140	eGFR			0.0434	[19]
		rs4145993		T	C	SCA	1140	eGFR			0.0352	[19]
		rs1434549		T	C	SCA	1140	eGFR			0.0109	[19]
	*NPRL3* ^9^	rs11248850	16p13.3	A	G	SCA	162	eGFR > 140	OR	17.69	0.013	[41]

Effect and effect size were noted whenever retrievable from the original study. eGFR values were always expressed as mL/min/1.73 m^2^. CAR—Central African Republic, CKD—chronic kidney disease, D—deletion, EA—effect allele, eGFR—estimated GFR, GFR—glomerular filtration rate, HR—hazard ratio, HRG—high-risk genotype, I—insertion, L—long GT-tandem repeat, LRG—low-risk genotype, N—number of patients included in the original study, na—not available, OA—other allele, OR—odds ratio, S—short GT-tandem repeat, Sbeta0—compound heterozygous hemoglobin S/beta^0^-thalassemia, SCA—sickle cell anemia, ^1^ method not defined in the paper, ^2^ eGFR < 15 mL/min/1.73 m^2^ or renal replacement therapy, ^3^ defined as 50% reduction in GFR or renal replacement therapy, ^4^ genetic risk profile defined as SCA patients with *APOL1* HRG, without alpha-thalassemia and with wild-type *BCL11* rs1427407 allele, ^5^ Outcome Modifying Genes in SCD cohort/Walk-Treatment of Pulmonary Hypertension and Sickle Cell Disease with Sidenafil Therapy cohort, ^6^ defined as a slope of ≥3 mL/min/1.73 m^2^, ^7^ eGFR ≥ 180 mL/min/1.73 m^2^ for 2–10 years and eGFR > 140 mL/min/1.73 m^2^ if >10 years, ^8^ defined as eGFR >130 mL/min/1.73 m^2^ for men and eGFR >140 mL/min/1.73 m^2^ for women, ^9^ only in combination with α^−3.7^ deletion, ^§^ additive/dominant model, * adjusted for age and sex, ** adjusted for age, sex, diabetes, systolic blood pressure, hydroxyurea therapy, angiotensin-converting-enzyme inhibitor or angiotensin receptor blocker use, body mass index, *** adjusted for age, sex, diabetes, systolic blood pressure, hydroxyurea therapy, angiotensin-converting-enzyme inhibitor or angiotensin receptor blocker use, body mass index, and urinary albumin-to-creatinine ratio.

## Data Availability

No new data were created or analyzed in this study. Data sharing is not applicable to this article.

## Supplementary Materials

The following are available online at https://www.mdpi.com/article/10.3390/ijms25105427/s1.
