# Supplementary material for: Systematic Review of Genetic Modifiers Associated with the Development and/or Progression of Nephropathy in Patients with Sickle Cell Disease
Source: Int J Mol Sci. 2024 May 16;25(10):5427. doi: 10.3390/ijms25105427 (PMC11121490; doi:10.3390/ijms25105427)
Supplement: Supplementary file 1 [file ijms-25-05427-s001.zip › ijms-2977087-supplementary.pdf]

Supplementary Table S1. Overview of the characteristics of included studies

Study	Study design	Genotype	Age	N	Ancestry	Gene	Outcome
Geard [9]	Case-control	SCA	all	413	Cameroon	APOL1, HMOX1, HBA1/HBA2	albuminuria, GFR
Garrett [17]	GWAS	all	adult	1078*	USA United Kingdom	n/a	albuminuria, GFR
Guasch [18]	Case-control	SCA	adult	76	Georgia	HBA1/HBA2, HbS haplotype	albuminuria
Nolan [19]	Case-control	SCA	all	1140	African-American	BMPR1B	GFR
Afenyi-Annan [20]	Case-control	SCA	adult	237	African-American	Duffy gene	albuminuria, GFR
Nebor [21]	Case-control	SCA	adult	189	Guadeloupe	HBA1/HBA2	albuminuria
Nebor [22]	Case-control	SCA	adult	212	Guadeloupe	Duffy gene	albuminuria, GFR
Ashley-Koch [23]	Case-control	all	adult	521	African-American	APOL1, MYH9	Proteinuria, GFR
Rocha [24]	Case-control	all	adult	84	Brazil	HbS haplotype	GFR, hyposthenuria, acidification deficit
Drasar [25]	Case-control	all	adult	272	African	Duffy gene	albuminuria, GFR
Saraf [26]	Case-control	all	adult	787**	USA United Kingdom	APOL1, MYH9, BMPR1B, HMOX1	albuminuria, GFR hemoglobinuria
Tantawy [27]	Case-control	SCA/Sbeta0	pediatric	51	Egypt	eNOS	albuminuria
Farawela [28]	Case-control	all	pediatric	100	Egypt	Duffy gene	albuminuria
Kormann [29]	Case-control	all	adult	152	Sub-Saharan West Indies	APOL1, HMOX1, GSTM1, GSTT1, GSTP1	albuminuria, GFR
Saraf [30]	Case-control	SCA/Sbeta0	adult	262	USA	APOL1, BCL11, HBA1/HBA2	albuminuria, GFR hemoglobinuria
Ilboudi [31]	Case-control	all	all	865	African-American, African, West Indies, Indian Ocean	PIEZO1	GFR
Amle [32]	Case-control	SCA	pediatric	60	India	VEGF	albuminuria
Zahr [33]	Case-control	SCA/Sbeta0	pediatric	291	African-American	APOL1	albuminuria

Belisario [34]	Case-control	SCA	pediatric	555	Brazil	HBA1/HBA2, HMOX1, ACE	albuminuria, GFR
Chinedu [35]	Case-control	SCA	adult	75	Brazil	HMOX1, BMPR1B	GFR
Ngo-Bitoungui [36]	Case-control	SCA	all	413	Cameroon	APOL1, HMOX1, 53 SNPs from GWAS	albuminuria, GFR
Rashkin [37]	Case-control	SCA	pediatric	288	African-American	APOL1, HMOX1, BCL11A, HBA1/HBA2, 45 SNPs from GWAS	albuminuria
Saraf [38]	Case-control	all	adult	299	African-American	21 genes based on mice studies	albuminuria, GFR hemoglobinuria
Adebayo [39]	Case-control	SCA	pediatric	326	DRC	APOL1, HMOX1	albuminuria, GFR
Brewin [40]	Case-control	SCA and HbSC	all	557	United Kingdom	HBA1/HBA2	albuminuria, GFR
Ndour [41]	Case-control	SCA	all	162	Senegal	Senegal haplotype, HBA1/HBA2, NPRL3, BCL11A	albuminuria, (tubular) proteinuria, GFR, hyposthenuria
Bhaskar [42]	Case-control	SCA	all	190	India	IL1RN	GFR
Schaefer [43]	Case-control	SCA	pediatric	197***	USA	APOL1, Duffy gene, eNOS4, CUBN	albuminuria
Schaefer [43]	GWAS	SCA	pediatric	187****	USA	n/a	GFR

Whenever the ancestry was not clear from the original study, we recorded where the study was performed. DRC – Democratic Republic of Congo, GFR – glomerular filtration rate, GWAS – genome-wide association study, n/a – not applicable, Sbeta0 – compound heterozygous hemoglobin S/beta^0-thalassemia, SCA – sickle cell anemia, SNP – single nucleotide polymorphism. *576 patients from the Outcome Modifying Genes in SCD cohort and 502 patients from the Walk-Treatment of Pulmonary Hypertension and Sickle Cell Disease with Sidenafil Therapy (Walk-PHaSST) cohort, **247 patients from the University of Illinois cohort and 540 patients from the Walk-PHaSST cohort, ***79 patients from the Hydroxyurea Study of Long-term Effects (HUSTLE) and 118 patients from the TCD With Transfusions Changing to Hydroxyurea (TWiTCH), ****62 patients in a discovery and 125 patients in a validation cohort.

Supplementary Table S2. PRISMA 2020 Checklist

Section and Topic	Item #	Checklist item	Location where item is reported
**TITLE**			
Title	1	Identify the report as a systematic review.	Page 1
**ABSTRACT**			
Abstract	2	See the PRISMA 2020 for Abstracts checklist.	Line 13-28
**INTRODUCTION**			
Rationale	3	Describe the rationale for the review in the context of existing knowledge.	Line 58-63
Objectives	4	Provide an explicit statement of the objective(s) or question(s) the review addresses.	Line 64-71
**METHODS**			
Eligibility criteria	5	Specify the inclusion and exclusion criteria for the review and how studies were grouped for the syntheses.	Line 88-94
Information sources	6	Specify all databases, registers, websites, organisations, reference lists and other sources searched or consulted to identify studies. Specify the date when each source was last searched or consulted.	Line 75-84
Search strategy	7	Present the full search strategies for all databases, registers and websites, including any filters and limits used.	Suppl. Table S3
Selection process	8	Specify the methods used to decide whether a study met the inclusion criteria of the review, including how many reviewers screened each record and each report retrieved, whether they worked independently, and if applicable, details of automation tools used in the process.	Line 95-104
Data collection process	9	Specify the methods used to collect data from reports, including how many reviewers collected data from each report, whether they worked independently, any processes for obtaining or confirming data from study investigators, and if applicable, details of automation tools used in the process.	Line 106-108 + line 571
Data items	10a	List and define all outcomes for which data were sought. Specify whether all results that were compatible with each outcome domain in each study were sought (e.g. for all measures, time points, analyses), and if not, the methods used to decide which results to collect.	Line 109-114
	10b	List and define all other variables for which data were sought (e.g. participant and intervention characteristics, funding sources). Describe any assumptions made about any missing or unclear information.	Line 109-114
Study risk of bias assessment	11	Specify the methods used to assess risk of bias in the included studies, including details of the tool(s) used, how many reviewers assessed each study and whether they worked independently, and if applicable, details of automation tools used in the process.	Line 117-118
Effect measures	12	Specify for each outcome the effect measure(s) (e.g. risk ratio, mean difference) used in the synthesis or presentation of results.	Table 1 + 2
Synthesis methods	13a	Describe the processes used to decide which studies were eligible for each synthesis (e.g. tabulating the study intervention characteristics and comparing against the planned groups for each synthesis (item #5)).	95-99
	13b	Describe any methods required to prepare the data for presentation or synthesis, such as handling of missing summary statistics, or data conversions.	na
	13c	Describe any methods used to tabulate or visually display results of individual studies and syntheses.	Line 108

Section and Topic	Item #	Checklist item	Location where item is reported
	13d	Describe any methods used to synthesize results and provide a rationale for the choice(s). If meta-analysis was performed, describe the model(s), method(s) to identify the presence and extent of statistical heterogeneity, and software package(s) used.	na
	13e	Describe any methods used to explore possible causes of heterogeneity among study results (e.g. subgroup analysis, meta-regression).	Not done
	13f	Describe any sensitivity analyses conducted to assess robustness of the synthesized results.	Not done
Reporting bias assessment	14	Describe any methods used to assess risk of bias due to missing results in a synthesis (arising from reporting biases).	Line 119-123
Certainty assessment	15	Describe any methods used to assess certainty (or confidence) in the body of evidence for an outcome.	na
**RESULTS**			
Study selection	16a	Describe the results of the search and selection process, from the number of records identified in the search to the number of studies included in the review, ideally using a flow diagram.	Figure 1
	16b	Cite studies that might appear to meet the inclusion criteria, but which were excluded, and explain why they were excluded.	Figure 1
Study characteristics	17	Cite each included study and present its characteristics.	Line 139-141 + Suppl. Table S1
Risk of bias in studies	18	Present assessments of risk of bias for each included study.	Line 404-409
Results of individual studies	19	For all outcomes, present, for each study: (a) summary statistics for each group (where appropriate) and (b) an effect estimate and its precision (e.g. confidence/credible interval), ideally using structured tables or plots.	Line 151-402+ table 1 and 2
Results of syntheses	20a	For each synthesis, briefly summarise the characteristics and risk of bias among contributing studies.	Line 151-402 + table 1 and 2
	20b	Present results of all statistical syntheses conducted. If meta-analysis was done, present for each the summary estimate and its precision (e.g. confidence/credible interval) and measures of statistical heterogeneity. If comparing groups, describe the direction of the effect.	Line 151-402 + table 1 and 2
	20c	Present results of all investigations of possible causes of heterogeneity among study results.	Line 151-402 + table 1 and 2
	20d	Present results of all sensitivity analyses conducted to assess the robustness of the synthesized results.	na
Reporting biases	21	Present assessments of risk of bias due to missing results (arising from reporting biases) for each synthesis assessed.	na
Certainty of	22	Present assessments of certainty (or confidence) in the body of evidence for each outcome assessed.	na

Section and Topic	Item #	Checklist item	Location where item is reported
evidence			
**DISCUSSION**			
Discussion	23a	Provide a general interpretation of the results in the context of other evidence.	Line 423-519
	23b	Discuss any limitations of the evidence included in the review.	Line 521-548
	23c	Discuss any limitations of the review processes used.	Line 521-548
	23d	Discuss implications of the results for practice, policy, and future research.	Line 551-565
**OTHER INFORMATION**			
Registration and protocol	24a	Provide registration information for the review, including register name and registration number, or state that the review was not registered.	Line 85-86
	24b	Indicate where the review protocol can be accessed, or state that a protocol was not prepared.	Line 85-86
	24c	Describe and explain any amendments to information provided at registration or in the protocol.	na
Support	25	Describe sources of financial or non-financial support for the review, and the role of the funders or sponsors in the review.	Line 574
Competing interests	26	Declare any competing interests of review authors.	Line 584
Availability of data, code and other materials	27	Report which of the following are publicly available and where they can be found: template data collection forms; data extracted from included studies; data used for all analyses; analytic code; any other materials used in the review.	Line 577-578

Supplementary Table S3. Search strategy

PubMed (including Medline)

Concept 1 = sickle cell disease patients

("Anemia, Sickle Cell"[Mesh:NoExp] OR "Hemoglobin SC Disease"[Mesh]) OR ("sickle cell"[tiab] OR "Hemoglobin S"[tiab] OR "Haemoglobin S"[tiab] OR "Hemoglobin SS"[tiab] OR "Haemoglobin SS"[tiab] OR "HbS Disease*"[tiab] OR "Hb S Disease*"[tiab] OR "Hb SS Disease*"[tiab] OR "HbSS Disease*"[tiab] OR "SS disease*"[tiab] OR "Hemoglobin SC"[tiab] OR "Haemoglobin SC"[tiab] OR "SC disease*"[tiab] OR "Hb SC Disease*"[tiab] OR "HbSC Disease*"[tiab] OR "sickle anaemia"[tiab] OR "sickle anemia"[tiab] OR "sickling disorder*"[tiab] OR "sickling disease*"[tiab] OR "sickle beta thalassaemia"[tiab] OR "sickle beta thalassemia"[tiab])

Concept 2 = genetic polymorphism/variant

("Genetic Association Studies"[Mesh] OR "Genes, Modifier"[Mesh] OR "Mutation"[Mesh] OR "Genetic Predisposition to Disease"[Mesh] OR "Genetic Variation"[Mesh:NoExp] OR "Polymorphism, Single Nucleotide"[Mesh] OR "Alleles"[Mesh] OR "Quantitative Trait Loci"[Mesh] OR "Haplotypes"[Mesh]) OR (GWAS[tiab] OR GWA[tiab] OR genome[tiab] OR gene[tiab] OR genes[tiab] OR genetic[tiab] OR mutation*[tiab] OR polymorphism*[tiab] OR SNP[tiab] OR SNPs [tiab] OR Allel*[tiab] OR "quantitative trait loc*"[tiab] OR QTL[tiab] OR hereditary[tiab] OR genotyp*[tiab] OR variant*[tiab] OR variation*[tiab] OR haplotyp*[tiab])

Concept 3 = nephropathy

("Kidney Diseases"[Mesh:NoExp] OR "Kidney Failure, Chronic"[Mesh] OR "Acidosis, Renal Tubular"[Mesh] OR "Glomerular Filtration Rate"[Mesh] OR "Proteinuria"[Mesh:NoExp] OR "Albuminuria"[Mesh] OR "Nephrotic Syndrome"[Mesh] OR "Hematuria"[Mesh] OR "Polyuria"[Mesh] OR "Enuresis"[Mesh]) OR (nephropath*[tiab] OR "kidney disease*"[tiab] OR "renal disease*"[tiab] OR "kidney disorder*"[tiab] OR "renal disorder*"[tiab] OR "kidney patholog*"[tiab] OR "renal patholog*"[tiab] OR "kidney insufficienc*"[tiab] OR "renal insufficienc*"[tiab] OR "kidney dysfunction*"[tiab] OR "renal dysfunction*"[tiab] OR "kidney impairment*"[tiab] OR "renal impairment*"[tiab] OR "kidney failure*"[tiab] OR "renal failure*"[tiab] OR ESRD[tiab] OR CKD[tiab] OR glomerulopath*[tiab] OR "glomerular disease*"[tiab] OR "glomerular dysfunction*"[tiab] OR "kidney tubular acidosis"[tiab] OR "renal tubular acidosis"[tiab] OR "renal tubule acidosis"[tiab] OR "kidney tubule acidosis"[tiab:~0] OR RTA[tiab] OR GFR[tiab] OR eGFR[tiab] OR "glomerular filtration rate"[tiab] OR "glomerulofiltration rate"[tiab] OR "glomerulus filtration rate"[tiab] OR hyperfiltration[tiab] OR hyposthenuria[tiab] OR proteinuria[tiab] OR albuminuria[tiab] OR nephrotic[tiab] OR "microalbuminuria"[tiab] OR "micro-albuminuria"[tiab] OR "macroalbuminuria"[tiab] OR "macro-albuminuria"[tiab] OR hematuria[tiab] OR haematuria[tiab] OR polyuria[tiab] OR enuresis[tiab] OR "bed-wetting"[tiab] OR "bedwetting "[tiab] OR "night-time urinary incontinence"[tiab] OR "night-time wetting"[tiab] OR "nighttime urinary incontinence"[tiab] OR "nighttime wetting"[tiab])

Embase (Embase.com)

Concept 1 = sickle cell disease patients

'sickle cell anemia'/de OR 'hemoglobin SC disease'/exp OR 'sickle cell beta thalassemia'/exp OR

('hemoglobin S' OR 'haemoglobin S' OR 'hemoglobin SS' OR 'haemoglobin SS' OR 'Hb S disease*' OR 'HbS disease*' OR 'Hb SS disease*' OR 'HbSS disease*' OR 'SS disease*' OR 'hemoglobin SC' OR 'haemoglobin SC' OR 'SC disease*' OR 'Hb SC disease*' OR 'HbSC disease*' OR 'sickle anaemia' OR 'sickle anemia' OR 'sickle cell' OR 'sickle beta thalassaemia' OR 'sickle beta thalassemia' OR 'sickling disorder*' OR 'sickling disease*'):ti,ab,kw

Concept 2 = genetic polymorphism/variant

'genetic association study'/exp OR 'modifier gene'/exp OR 'gene mutation'/exp OR 'genetic predisposition'/exp OR 'single nucleotide polymorphism'/exp OR 'allele'/de OR 'quantitative trait locus'/exp OR 'haplotype'/exp OR ('GWAS' OR 'GWA' OR 'genome' OR 'gene' OR 'genes' OR 'genetic' OR 'mutation*' OR 'polymorphism*' OR 'SNP' OR 'SNPs' OR 'allel*' OR 'quantitative trait loc*' OR 'QTL' OR 'hereditary' OR 'genotyp*' OR 'variant*' OR 'variation*' OR 'haplotyp*'):ti,ab,kw

Concept 3 = nephropathy

'kidney disease'/de OR 'glomerulopathy'/de OR 'kidney dysfunction'/de OR 'glomerular dysfunction'/exp OR 'kidney failure'/de OR 'chronic kidney failure'/exp OR 'end stage renal disease'/exp OR 'kidney tubule acidosis'/exp OR 'glomerulus filtration rate'/exp OR 'proteinuria'/exp OR 'nephrotic syndrome'/exp OR 'hematuria'/exp OR 'polyuria'/exp OR 'enuresis'/exp OR ('nephropath*' OR 'kidney disease*' OR 'kidney disorder*' OR 'kidney patholog*' OR 'renal patholog*' OR 'renal disease*' OR 'renal disorder*' OR 'glomerulopath*' OR 'glomerular disease*' OR 'glomerular dysfunction*' OR 'kidney dysfunction*' OR 'renal dysfunction*' OR 'kidney failure*' OR 'renal failure*' OR 'ESRD' OR 'CKD' OR 'renal insufficienc*' OR 'kidney insufficienc*' OR 'kidney impairment*' OR 'renal impairment*' OR 'kidney tubular acidosis' OR 'renal tubular acidosis' OR 'renal tubule acidosis' OR 'kidney tubule acidosis' OR 'RTA' OR 'GFR' OR 'eGFR' OR 'glomerular filtration rate' OR 'glomerulofiltration rate' OR 'glomerulus filtration rate' OR 'hyperfiltration' OR 'hyposthenuria' OR 'proteinuria' OR 'albuminuria' OR 'nephrotic' OR 'microalbuminuria' OR 'micro-albuminuria' OR 'macroalbuminuria' OR 'macro-albuminuria' OR 'hematuria' OR 'haematuria' OR 'enuresis' OR 'bed-wetting' OR 'bedwetting' OR 'night-time urinary incontinence' OR 'night-time wetting' OR 'nighttime urinary incontinence' OR 'nighttime wetting' OR 'polyuria'):ti,ab,kw

Web of Science (Core Collection)

Science Citation Index Expanded

(SCI-EXPANDED)--1955-present

Social Sciences Citation Index

(SSCI)--1956-present

Arts & Humanities Citation Index

(AHCI)--1975-present

Conference Proceedings Citation Index – Science

(CPCI-S)--1990-present

Conference Proceedings Citation Index – Social Science & Humanities

(CPCI-SSH)--1990-present

Emerging Sources Citation Index

(ESCI)--2018-present

Concept 1 = sickle cell disease patients

TS=("sickle cell" OR "Hemoglobin S" OR "Haemoglobin S" OR "Hemoglobin SS" OR "Haemoglobin SS" OR "HbS Disease*" OR "Hb S Disease*" OR "Hb SS Disease*" OR "HbSS Disease*" OR "SS disease*" OR "Hemoglobin SC" OR "Haemoglobin SC" OR "SC disease*" OR "Hb SC Disease*" OR "HbSC Disease*" OR "sickle anaemia" OR "sickle anemia" OR "sickling disorder*" OR "sickling disease*" OR "sickle beta thalassaemia" OR "sickle beta thalassemia")

Concept 2 = genetic polymorphism/variant

TS=("GWAS" OR "GWA" OR "genome" OR "gene" OR "genes" OR "genetic" OR "mutation*" OR "polymorphism*" OR "SNP" OR "SNPs" OR "Allel*" OR "quantitative trait loc*" OR "QTL" OR "hereditary" OR "genotyp*" OR "variant*" OR "variation*" OR "haplotyp*")

Concept 3 = nephropathy

TS=("nephropath*" OR "kidney disease*" OR "renal disease*" OR "kidney disorder*" OR "renal disorder*" OR "kidney patholog*" OR "renal patholog*" OR "kidney insufficienc*" OR "renal insufficienc*" OR "kidney dysfunction*" OR "renal dysfunction*" OR "kidney impairment*" OR "renal impairment*" OR "kidney failure*" OR "renal failure*" OR "ESRD" OR "CKD" OR "glomerulopath*" OR "glomerular disease*" OR "glomerular dysfunction*" OR "kidney tubular acidosis" OR "renal tubular acidosis" OR "renal tubule acidosis" OR "kidney tubule acidosis" OR "RTA" OR "GFR" OR "eGFR" OR "glomerular filtration rate" OR "glomerulofiltration rate" OR "glomerulus filtration rate" OR "hyperfiltration" OR "hyposthenuria" OR "proteinuria" OR "albuminuria" OR "nephrotic" OR "microalbuminuria" OR "micro-albuminuria" OR "macroalbuminuria" OR "macro-albuminuria" OR "hematuria" OR "haematuria" OR "polyuria" OR "enuresis" OR "bed-wetting" OR "bedwetting " OR "night-time urinary incontinence" OR "night-time wetting" OR "nighttime urinary incontinence" OR "nighttime wetting")

<u>CENTRAL (Cochrane Library)</u>

Concept 1 = sickle cell disease patients

#1: [mh ^"Anemia, Sickle Cell"] OR [mh "Hemoglobin SC Disease"]

#2: ("sickle cell" OR "Hemoglobin S" OR "Haemoglobin S" OR "Hemoglobin SS" OR "Haemoglobin SS" OR (HbS NEXT Disease*) OR ("Hb S" NEXT Disease*) OR ("Hb SS" NEXT Disease*) OR (HbSS NEXT Disease*) OR (SS NEXT disease*) OR "Hemoglobin SC" OR "Haemoglobin SC" OR (SC NEXT disease*) OR ("Hb SC" NEXT Disease*) OR (HbSC NEXT Disease*) OR "sickle anaemia" OR "sickle anemia" OR (sickling NEXT disorder*) OR (sickling NEXT disease*) OR "sickle beta thalassaemia" OR "sickle beta thalassemia"):ti,ab,kw

#3: #1 OR #2

Concept 2 = genetic polymorphism/variant

#4: [mh "Genetic Association Studies"] OR [mh "Genes, Modifier"] OR [mh "Mutation"] OR [mh "Genetic Predisposition to Disease"] OR [mh ^"Genetic Variation"] OR [mh "Polymorphism, Single Nucleotide"] OR [mh "Alleles"] OR [mh "Quantitative Trait Loci"] OR [mh "Haplotypes"]

#5: ("GWAS" OR "GWA" OR "genome" OR "gene" OR "genes" OR (mutation*) OR (polymorphism*) OR "SNP" OR "SNPs" OR (Allel*) OR ("quantitative trait" NEXT loc*) OR "QTL" OR "genetic" OR "hereditary" OR (genotyp*) OR (variant*) OR (variation*) OR (haplotyp*)):ti,ab,kw

#6: #4 OR #5

Concept 3 = nephropathy

#7: [mh ^"Kidney Diseases"] OR [mh "Kidney Failure, Chronic"] OR [mh "Acidosis, Renal Tubular"] OR [mh "Glomerular Filtration Rate"] OR [mh ^"Proteinuria"] OR [mh "Albuminuria"] OR [mh "Nephrotic Syndrome"] OR [mh "Hematuria"] OR [mh "Polyuria"] OR [mh "Enuresis"]

#8: ((nephropath*) OR (kidney NEXT disease*) OR (renal NEXT disease*) OR (kidney NEXT disorder*) OR (renal NEXT disorder*) OR (kidney NEXT patholog*) OR (renal NEXT patholog*) OR (glomerulopath*) OR (glomerular NEXT disease*) OR (glomerular NEXT dysfunction*) OR (kidney NEXT failure*) OR (renal NEXT failure*) OR "ESRD" OR "CKD" OR (renal NEXT insufficienc*) OR (kidney NEXT insufficienc*) OR (kidney NEXT dysfunction*) OR (renal NEXT dysfunction*) OR (kidney NEXT impairment*) OR (renal NEXT impairment*) OR "kidney tubular acidosis" OR "renal tubular acidosis" OR "renal tubule acidosis" OR "kidney tubule acidosis" OR "RTA" OR "GFR" OR "eGFR" OR "glomerular filtration rate" OR "glomerulofiltration rate" OR "glomerulus filtration rate" OR "hyperfiltration" OR "hyposthenuria" OR "proteinuria" OR "albuminuria" OR "nephrotic" OR "microalbuminuria" OR "micro-albuminuria" OR "macroalbuminuria" OR "macro-albuminuria" OR "hematuria" OR "haematuria" OR "enuresis" OR "bed-wetting" OR "bedwetting" OR "night-time urinary incontinence" OR "night-time wetting" OR "nighttime urinary incontinence" OR "nighttime wetting" OR "polyuria"):ti,ab,kw

#9: #7 OR #8

#10: #3 AND #6 AND #9

__Scopus__

Concept 1 = sickle cell disease patients

TITLE-ABS("sickle cell" OR "Hemoglobin S" OR "Haemoglobin S" OR "Hemoglobin SS" OR "Haemoglobin SS" OR "HbS Disease*" OR "Hb S Disease*" OR "Hb SS Disease*" OR "HbSS Disease*" OR "SS disease*" OR "Hemoglobin SC" OR "Haemoglobin SC" OR "SC disease*" OR "Hb SC Disease*" OR "HbSC Disease*" OR "sickle anaemia" OR "sickle anemia" OR "sickling disorder*" OR "sickling disease*" OR "sickle beta thalassaemia" OR "sickle beta thalassemia") OR AUTHKEY("sickle cell" OR "Hemoglobin S" OR "Haemoglobin S" OR "Hemoglobin SS" OR "Haemoglobin SS" OR "HbS Disease*" OR "Hb S Disease*" OR "Hb SS Disease*" OR "HbSS Disease*" OR "SS disease*" OR "Hemoglobin SC" OR "Haemoglobin SC" OR "SC disease*" OR "Hb SC Disease*" OR "HbSC Disease*" OR "sickle anaemia" OR "sickle anemia" OR "sickling disorder*" OR "sickling disease*" OR "sickle beta thalassaemia" OR "sickle beta thalassemia")

Concept 2 = genetic polymorphism/variant

TITLE-ABS("GWAS" OR "GWA" OR "genome" OR "gene" OR "genes" OR "genetic" OR "mutation*" OR "polymorphism*" OR "SNP" OR "SNPs" OR "Allel*" OR "quantitative trait loc*" OR "QTL" OR "hereditary" OR "genotyp*" OR "variant*" OR "variation*" OR "haplotyp*") OR AUTHKEY("GWAS" OR "GWA" OR "genome" OR "gene" OR "genes" OR "genetic" OR "mutation*" OR "polymorphism*" OR "SNP" OR "SNPs" OR "Allel*" OR "quantitative trait loc*" OR "QTL" OR "hereditary" OR "genotyp*" OR "variant*" OR "variation*" OR "haplotyp*")

Concept 3 = nephropathy

TITLE-ABS("nephropath*" OR "kidney disease*" OR "renal disease*" OR "kidney disorder*" OR "renal disorder*" OR "kidney patholog*" OR "renal patholog*" OR "kidney insufficienc*" OR "renal insufficienc*" OR "kidney dysfunction*" OR "renal dysfunction*" OR "kidney impairment*" OR "renal impairment*" OR "kidney failure*" OR "renal failure*" OR "ESRD" OR "CKD" OR "glomerulopath*" OR "glomerular disease*" OR "glomerular dysfunction*" OR "kidney tubular acidosis" OR "renal tubular acidosis" OR "renal tubule acidosis" OR "kidney tubule acidosis" OR "RTA" OR "GFR" OR "eGFR" OR "glomerular filtration rate" OR "glomerulofiltration rate" OR "glomerulus filtration rate" OR "hyperfiltration" OR "hyposthenuria" OR "proteinuria" OR "albuminuria" OR "nephrotic" OR "microalbuminuria" OR "micro-albuminuria" OR "macroalbuminuria" OR "macro-albuminuria" OR "hematuria" OR "haematuria" OR "polyuria" OR "enuresis" OR "bed-wetting" OR "bedwetting " OR "night-time urinary incontinence" OR "night-time wetting" OR "nighttime urinary incontinence" OR "nighttime wetting") OR AUTHKEY("nephropath*" OR "kidney disease*" OR "renal disease*" OR "kidney disorder*" OR "renal disorder*" OR "kidney patholog*" OR "renal patholog*" OR "kidney insufficienc*" OR "renal insufficienc*" OR "kidney dysfunction*" OR "renal dysfunction*" OR "kidney impairment*" OR "renal impairment*" OR "kidney failure*" OR "renal failure*" OR "ESRD" OR "CKD" OR "glomerulopath*" OR "glomerular disease*" OR "glomerular dysfunction*" OR "kidney tubular acidosis" OR "renal tubular acidosis" OR "renal tubule acidosis" OR "kidney tubule acidosis" OR "RTA" OR "GFR" OR "eGFR" OR "glomerular filtration rate" OR "glomerulofiltration rate" OR "glomerulus filtration rate" OR "hyperfiltration" OR "hyposthenuria" OR "proteinuria" OR "albuminuria" OR "nephrotic" OR "microalbuminuria" OR "micro-albuminuria" OR "macroalbuminuria" OR "macro-albuminuria" OR "hematuria" OR "haematuria" OR "polyuria" OR "enuresis" OR "bed-wetting" OR "bedwetting " OR "night-time urinary incontinence" OR "night-time wetting" OR "nighttime urinary incontinence" OR "nighttime wetting")
